# The *BAG2* and *BAG6* Genes Are Involved in Multiple Abiotic Stress Tolerances in *Arabidopsis Thaliana*

**DOI:** 10.3390/ijms22115856

**Published:** 2021-05-29

**Authors:** Muhammad Arif, Zitong Li, Qiong Luo, Luhua Li, Yuequan Shen, Shuzhen Men

**Affiliations:** 1 Tianjin Key Laboratory of Protein Sciences, Department of Plant Biology and Ecology, College of Life Sciences, Nankai University, Tianjin 300071, China; arifbiotech144@gmail.com (M.A.); zitongli@mail.nankai.edu.cn (Z.L.); qiongluo2016@mail.nankai.edu.cn (Q.L.); luhua_li@163.com (L.L.); 2 State Key Laboratory of Medicinal Chemical Biology, Nankai University, 94 Weijin Road, Tianjin 300071, China; yshen@nankai.edu.cn; 3 Tianjin Key Laboratory of Protein Sciences, Department of Biochemistry and Molecular Biology, College of Life Sciences, Nankai University, Tianjin 300071, China

**Keywords:** Bcl-2 associated athanogene (BAG), *BAG2*, *BAG6*, Arabidopsis, drought stress, heat stress, abscisic acid (ABA), reactive oxygen species (ROS)

## Abstract

The BAG proteins are a family of multi-functional co-chaperones. In plants, BAG proteins were found to play roles both in abiotic and biotic stress tolerance. However, the function of Arabidopsis *BAG2* remains largely unknown, whereas *BAG6* is required for plants’ defense to pathogens, although it remains unknown whether *BAG6* is involved in plants’ tolerance to abiotic stresses. Here, we show that both *BAG2* and *BAG6* are expressed in various tissues and are upregulated by salt, mannitol, and heat treatments and by stress-related hormones including ABA, ethylene, and SA. Germination of *bag2*, *bag6* and *bag2 bag6* seeds is less sensitive to ABA compared to the wild type (WT), whereas *BAG2* and *BAG6* overexpression lines are hypersensitive to ABA. *bag2*, *bag6*, and *bag2 bag6* plants show higher survival rates than WT in drought treatment but display lower survival rates in heat-stress treatment. Consistently, these mutants showed differential expression of several stress- and ABA-related genes such as *RD29A*, *RD29B*, *NCED3* and *ABI4* compared to the WT. Furthermore, these mutants exhibit lower levels of ROS after drought and ABA treatment but higher ROS accumulation after heat treatment than the WT. These results suggest that *BAG2* and *BAG6* are negatively involved in drought stress but play a positive role in heat stress in Arabidopsis.

## 1. Introduction

Bcl-2 (B cell lymphorma 2)-associated athanogene (BAG)-family proteins were first identified by screening for Bcl-2-interacting proteins [1,2]. BAG proteins interact physically and functionally with the Bcl-2 protein to suppress cell death [1,2]. In mammals, there are six BAG proteins (BAG1–BAG6) that contain a conserved BAG domain at their C-terminal [3]. The BAG domain is approximately 110 amino acids in length and forms three α helices in three-dimensional structures [3,4]. Through the BAG domain, BAG proteins can interact with the ATPase domain of heat shock protein 70 (both constitutive Hsc70 and inducible Hsp70) and modulate the chaperone activity of these hsc70/hsp70 proteins [5]. At their N-terminal, some BAG proteins contain an ubiquitin-like domain (UBL) and/or several other motifs such as proline rich repeat (PXXP) and WW domain [3]. These domains/motifs enable the BAG proteins to interact with diverse binding partners that are involved in protein degradation, cell migration, cell proliferation, and apoptosis [3,6]. BAG family proteins also participate in Parkin-dependent mitophagy processes [7].

The BAG proteins are evolutionarily conserved with homologs present in fungi, plants and animals [8,9,10,11,12,13]. In plants, BAG proteins perform functions in apoptosis, senescence, and responses to biotic and abiotic stress [9,10,11,14,15,16,17]. Seven *BAG* genes were identified in the model plant *Arabidopsis thaliana* (Arabidopsis), ranging from *BAG1* to *BAG7* [9,10,11]. The AtBAG1–4 proteins contain a BAG domain and an UBL domain, whereas the AtBAG5–7 proteins contain an IQ motif, which preferentially binds to Ca^2+^-free calmodulin (CaM), in addition to a BAG domain [4,9,10,11,12,15]. Phylogenetic analysis by MEGA7 software clearly revealed that the plant BAG family proteins can be divided into two subgroups: Group I including the BAG1-4 proteins, and Group II including the BAG5-7 proteins (Figure S1). Arabidopsis BAG proteins have different subcellular localizations. AtBAG1–3 are mainly localized to the cytosol [18]; AtBAG4 is present in both cytosol and nucleus [18]; AtBAG5 is localized to the mitochondria [15]; AtBAG6 is localized to the nucleus [19]; and AtBAG7 resides in the endoplasmic reticulum (ER) [18,20]. These distinct subcellular localizations suggest that AtBAG proteins are functionally distinct.

AtBAG1 was found to be upregulated in *plastid protein import2* (*ppi2*) mutant plants, implying that it has a role in proteasomal degradation of unimported plastid proteins [18]. Further research showed that indeed, AtBAG1 interacts with the cytosolic Hsc70-4 chaperone through its BAG domain to cooperatively mediate proteasomal degradation of misfolded cytosolic proteins and unimported plastid proteins [18]. Physiologically, *AtBAG1*-overexpressing transgenic seedlings were more sensitive to salt treatment, suggesting that *AtBAG1* might be involved in plants’ response to abiotic stress [18]. It has been reported that under normal growth conditions the *atbag2* mutant exhibited slightly better growth than the WT, implying that AtBAG2 might participate in plants PCD or response to environmental stresses [4]. *AtBAG3* expression was upregulated by salt stress and by the stress-related hormones ABA and ACC, but downregulated by cold stress [15]. However, so far the physiological functions of *AtBAG2* and *AtBAG3* remain largely unknown.

Among the plant *BAG* genes, *BAG4* is one of the most studied. Overexpression of *AtBAG4* in tobacco plants confers tolerance to UVB irradiation and cold, oxidant and salt treatments [10]. In contrast, the *atbag4* mutant is more sensitive to salt treatment [11]. These results clearly indicate that AtBAG4 plays a role in plant response to abiotic stress. In rice, overexpression of *OsBAG4* enhances resistance to broad-spectrum disease, indicating that OsBAG4 functions as a positive regulator of disease resistance [21]. Furthermore, a rice E3 ubiquitin ligase EBR1 (enhanced blight and blast resistance 1) targets OsBAG4 for proteasome degradation; hence EBR1 and OsBAG4 form a module to balance growth and immune response [21]. Recently, AtBAG4 was found to interact with the guard cell potassium influx channel protein KAT1 to regulate stomatal movement [22]. Rice OsHKT1;5 (HIGH-AFFINITY POTASSIUM TRANSPORTER 1;5) is a Na^+^-selective transporter that maintains cellular Na^+^/K^+^ homeostasis under salinity stress [23]. Recent research shows that OsBAG4 functions as a bridge between OsMYB106 (a MYB transcription factor) and OsSUVH7 (a DNA methylation reader) to facilitate OsMYB106 binding to the *OsHKT1;5* promoter, thereby collaboratively regulating *OsHKT1;5* expression [24]. This research provides mechanisms for BAG4 function during plant responses to biotic and abiotic stress.

AtBAG5 contains both an IQ motif that can bind calmodulin (CaM) and a BAG domain capable of binding Hsc70/Hsp70 [15]. Our previous structural and biochemical studies revealed that Ca^2+^-free CaM (exhibit a closed conformation) and Hsc70 bind AtBAG5 independently, whereas Ca^2+^–saturated CaM (adopts an open conformation) and Hsc70 bind AtBAG5 with negative cooperativity [15]. We further demonstrated that AtBAG5 physiologically acts as a hub linking calcium signaling and the Hsc70 chaperone to regulate leaf senescence [15,25].

AtBAG6 also contains an IQ motif that has higher binding affinity for Ca^2+^-free CaM than the Ca^2+^–saturated CaM [26]. *AtBAG6* expression is upregulated by plant PCD inducing agent and overexpression of *AtBAG6* activates cell death [26]. Moreover, both the IQ motif and the BAG domain are required for AtBAG6-activated cell death [26]. By contrast, the *atbag6* mutant exhibits enhanced susceptibility to a plant fungal pathogen *Botrytis cinerea* [11]. These results suggest that AtBAG6 plays a role in plant response to biotic stress. Further research revealed the molecular mechanism for AtBAG6-mediated pathogen resistance [27]. Upon pathogen infection, AtBAG6 protein was cleaved by an aspartyl protease APCB1 (Aspartyl Protease Cleaving BAG) at a caspase-1 cleavage site (LATD) downstream of its BAG domain [27]. Furthermore, AtBAG6 cleavage triggers autophagy and plant defense [27]. *AtBAG6* transcription is also induced by stress-related plant hormones such as SA and ABA, and by heat stress [17,19]. AtBAG6 protein level is also significantly upregulated under heat stress [28]. The *atbag6* mutant is sensitive to non-acclimated severe heat stress (45 °C for 30 min) [17]. However, another report found that the *atbag6* mutant exhibited mild thermotolerance to non-acclimated heat stress (45 °C for 30 min) compared to the WT plants [28]. In acclimated heat stress treatment (38 °C for 2 h, followed by 45 °C for 1-3 h), the *atbag6* mutant showed no different phenotype from those of the WT [19]. However, *AtBAG6* mutation could increase the thermotolerance of the *fes1a* mutant [19]. Fes1A is a nucleotide exchange factor (NEF) of the HSP70 chaperone, and its mutation causes degradation of the HSP70 protein and leads to reduced tolerance to heat stress [29,30].

AtBAG7 resides in the ER and binds to the ER localized Hsp70 proteins to regulate unfolded protein response (UPR) during heat and cold tolerance [20]. Further study demonstrated that AtBAG7 may play a role in ER-nucleus retrograde signaling in plant responses to heat stress [31]. Upon heat treatment, AtBAG7 is sumoylated and proteolytically cleaved at the Ile^378^ site of the transmembrane domain in the C-terminus, then the cleaved N-terminus of AtBAG7 is translocated from the ER to the nucleus, where it interacts with the WRKY29 transcription factor to regulate expression of heat response genes [31]. It has been reported that salt treatment downregulated expression of *AtBAG6* and *AtBAG7*, but ACC treatment could reverse the effect of salinity [32]. Although *BAG6* was found to play a significant role in biotic (pathogens) and abiotic stresses (heat), it remains unknown whether BAG6 is involved in plants’ response to other stressors or not.

Here, we have focused on characterization of the functions of *BAG2* and *BAG6* in Arabidopsis. We first analyzed the expression patterns of the *BAG2* and *BAG6* genes during Arabidopsis development and in response to various hormones and abiotic stresses. Then, we analyzed the *bag2*, *bag6*, and *bag2 bag6* mutant phenotypes grown under normal or stressed conditions. Our results show that both *BAG2* and *BAG6* genes are expressed in various tissues in Arabidopsis. Expression of both *BAG2* and *BAG6* genes is induced by salt, mannitol, and heat treatments and by stress-related hormones including ABA, ACC (an ethylene precursor) and SA, while *BAG6* is additionally induced by JA. Germination of the *bag2* and *bag6* single and *bag2 bag6* double mutant seeds are less sensitive to ABA than that of the WT. Both the *bag2* and *bag6* single mutant and *bag2 bag6* double mutant showed higher survival rates than the WT in drought treatment but displayed lower survival rates in 45 °C heat stress experiments. Consistently, these mutants displayed differential transcription levels of stress-related genes such as *RD29A* and *RD29B*, ABA biosynthesis gene *NCED3*, and ABA response gene *ABI4* compared to the WT in response to ABA treatment. Finally, when compared to WT seedlings, the mutants exhibited higher content of reactive oxygen species (ROS) after heat stress but lower ROS levels after drought and ABA treatment.

## 2. Results

### 2.1. BAG2 and BAG6 Expression during Arabidopsis Development

**Figure 1:**
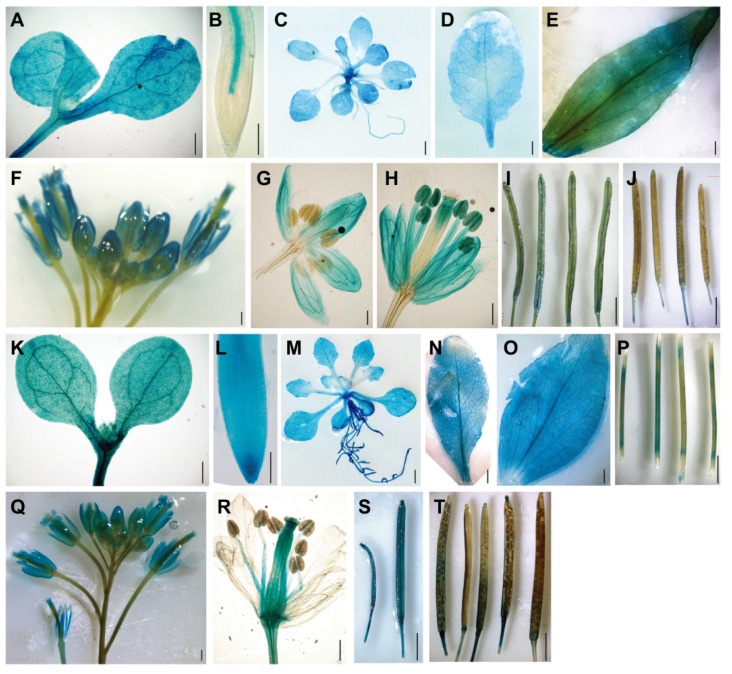
Expression patterns of the Arabidopsis *BAG2* and *BAG6* genes. (**A**–**J**) Expression of *ProBAG2:GUS* in 5-day-old seedlings cotyledons and hypocotyl (**A**), root tip (**B**), 2-week-old plant (**C**), rosette leaf (**D**), cauline leaf (**E**), inflorescence (**F**), flower bud (**G**), opened flower (**H**), young siliques (**I**), and mature siliques (**J**); (**K**–**T**) Expression of *ProBAG6:GUS* in cotyledons and hypocotyl (**K**) and root tip (**L**) of 5-day-old seedling, 2-week-old plant (**M**), rosette leaf (**N**), cauline leaf (**O**), stems (**P**), inflorescence (**Q**), opened flower (**R**), young siliques (**S**), and mature siliques (**T**). Bars = 100 μm in (**B**,**L**), 400 μm in (**A**,**F**–**H**,**K**,**Q**,**R**) and 2 mm in (**C**–**E**,**I**,**J**,**M**–**P**,**S**,**T**).

To determine expression patterns of the *BAG2* and *BAG6* genes, we generated Arabidopsis transgenic lines expressing the *GUS* reporter gene under control of the promoter fragments of the *BAG2* and *BAG6* genes, respectively (*ProBAG2:GUS* and *ProBAG6:GUS*) and analyzed the expression of the *GUS* reporter gene in various tissues. Strong GUS staining was observed in most tissues throughout the *ProBAG2:GUS* and *ProBAG6:GUS* transgenic plants (Figure 1A–T). In 7-day-old seedlings, *ProBAG2:GUS* activity is strongly detected in cotyledons, hypocotyl, and root vascular tissues, with the strongest signal at the lower part of the hypocotyl (Figure 1A,B), while *ProBAG6:GUS* expression is strongly detected in all of the tissues of the whole seedling (Figure 1K,L). In 2-week-old plants and bolting plants, both *ProBAG2:GUS* and *ProBAG6:GUS* are highly expressed in rosette leaves and cauline leaves (Figure 1C–E,M–O), with *ProBAG6:GUS* showing additional *GUS* activity in stems (Figure 1P). In flowers, both *ProBAG2:GUS* and *ProBAG6:GUS* are expressed in sepals, anther filaments, pollen grains, and style (Figure 1H,Q,R), with *ProBAG6:GUS* showing additional expression in ovules (Figure 1R). In siliques, both *ProBAG2:GUS* and *ProBAG6:GUS* are expressed in young siliques with *ProBAG6:GUS* showing stronger *GUS* signals (Figure 1I,S), while in old siliques both genes’ expressions are very weak (Figure 1J,T).

### 2.2. BAG2 and BAG6 Expression Responses to Abiotic Stress and Plant Hormones

**Figure 2:**
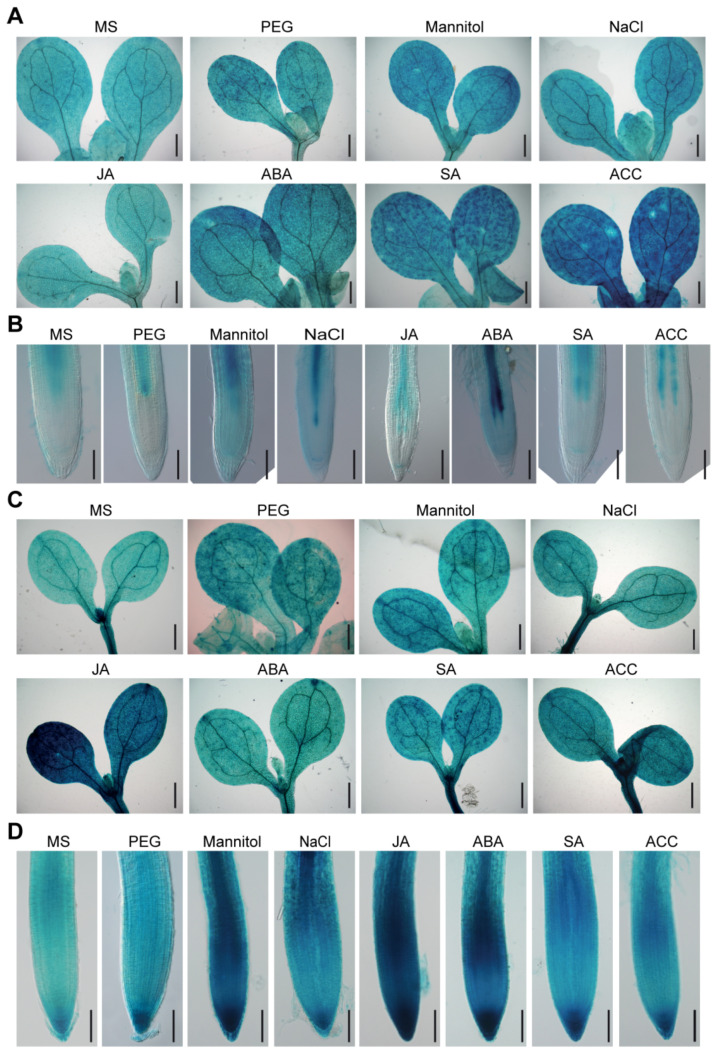
Expression of *ProBAG2:GUS* and *ProBAG6:GUS* in response to various abiotic stresses and hormones. Five-day-old *ProBAG2:GUS* (**A**,**B**) and *ProBAG6:GUS* (c and d) seedlings were transferred to Murashige and Skoog (MS) media (mock) supplemented with 5% PEG, 300 mM mannitol, 200 mM NaCl, 10 μM JA, 10 μM ABA, 5 mM SA and 10 μM ACC and treated for 24 h, then were collected for GUS staining. Shown are representative images of shoots (**A**,**C**) and root tips (**B**,**D**) of three independent experiments. Bars = 400 μm in (**A**,**C**) and 100 μm in (**B**,**D**).

We further investigated whether *BAG2* and *BAG6* expression is regulated by abiotic stressors or plant hormones. The results show that *ProBAG2:GUS* expression is strongly increased by mannitol, salt (NaCl), heat and ABA treatments (Figure 2A,B and Figure S2A) and its expression is also slightly increased in response to other stressors and hormones such as PEG, SA and ACC, respectively. Similarly, *ProBAG6:GUS* showed significant enhanced *GUS* staining in response to JA, ABA, mannitol, salt, and heat treatment, and showed a slight increase in response to ACC, PEG and SA (Figure 2C,D and Figure S2B–D). A previous report had analyzed *AtBAG* genes expression response to abiotic stressors such as cold, heat, mannitol, and salt and hormones such as ABA, ACC, MeJA, and SA by reverse transcription quantitative PCR (RT-qPCR) and showed that *AtBAG2* transcription is upregulated by ABA but downregulated by ACC, whereas *AtBAG6* transcription is upregulated by SA and ABA [17]. As to response to abiotic stresses, *AtBAG2* transcription is upregulated by salt but downregulated by cold, whereas *AtBAG6* transcription is upregulated by heat and salt treatment [17]. Together, these results suggest that *AtBAG2* and *AtBAG6* genes are involved in Arabidopsis responses to environmental stress.

### 2.3. BAG2 and BAG6 Genes Are Involved in Arabidopsis Responses to ABA and Drought Treatment

**Figure 3:**
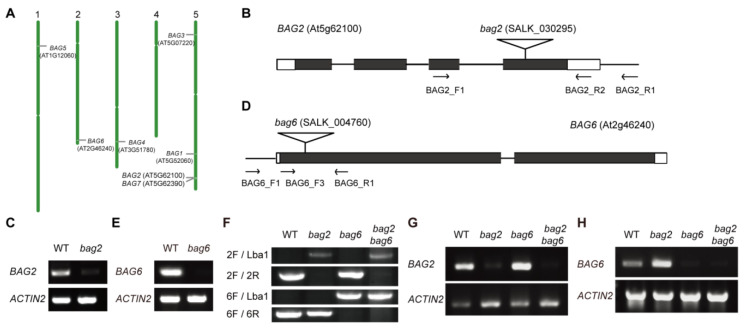
Chromosomal locations of the seven *BAG* genes. (**A**) The location of the gene is marked with short lines. White mark is the centromere of the chromosome. There are 7 *BAG* genes that are distributed on 5 chromosomes. *AtBAG2* is located on chromosome 5 and *AtBAG6* is located on chromosome 2. Insertion sites and transcription analysis of the *bag2* and *bag6* mutants; (**B**,**D**) Structures of the *AtBAG2* (**B**) and the *AtBAG6* (**D**) genes with T-DNA insertion sites. White boxes at left and right borders represent UTR regions, black boxes indicate exons, lines between black boxes represent introns, flags indicate the T-DNA insertion sites, and arrows indicate the positions of primers used for PCR verification of the insertions and for RT-PCR; (**C**,**E**) RT-PCR analysis of transcription levels of *AtBAG2* (**C**) and *AtBAG6* (**E**) genes in WT and *bag2* or *bag6* mutants. The *ACTIN2* gene was used as the internal control. Total RNA was extracted from 7-day-old seedlings. The presented images in (**C**,**E**) were cropped and the original, full-length gel images were provided in the additional file Figure S3; (**F**) Genotyping results of *bag2, bag6,* and *bag2 bag6* mutants; (**G**,**H)** RT-PCR analysis of transcription levels of *AtBAG2* and *AtBAG6* genes in *bag2, bag6,* and *bag2 bag6* mutants. The *ACTIN2* gene was used Eas the internal control.

To explore the function of the *BAG2* and *BAG6* genes, we analyzed localization of the genes on Arabidopsis chromosomes, and found that chromosome 5 has the highest number of genes, followed by others. *AtBAG2* is located on chromosome 5 (chr.5) and *AtBAG6* is located on chromosome 2 (chr.2) (Figure 3A). The transfer DNA (T-DNA) insertion mutants were isolated for *BAG2* and *BAG6* within the fourth exon (SALK_030295, *bag2*; Figure 3B) and within the first exon (SALK_004760, *bag6*; Figure 3D), respectively. Homozygous *bag2* and *bag6* mutants were crossed to generate the *bag2 bag6* double mutant (Figure 3F). The *bag2* and *bag6* mutants had been reported previously [4,28]. RT-PCR analyses revealed that weak *BAG2* transcript was detected in the homozygous *bag2* and *bag2 bag6* mutants (Figure 3C,G and Figure S3A,B) and no *BAG6* transcript was detectable in the homozygous *bag6* and *bag2 bag6* mutants (Figure 3E,H and Figure S3C,D), indicating that the *bag2* mutant is a knockdown allele and the *bag6* mutant is a null allele. Under normal growth conditions, the *bag2* and *bag6* single and *bag2 bag6* double mutants exhibited slightly larger rosette size than the WT (Figure S4).

**Figure 4:**
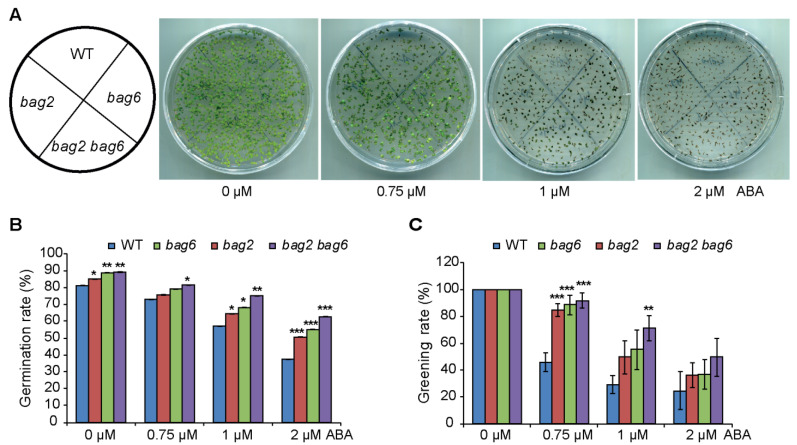
*bag2* and *bag6* mutants are less sensitive to ABA than the WT. (**A**–**C**) Seed germination rate and greening rate of WT, *bag2* and *bag6* single, and *bag2 bag6* double mutants. Sterilized seeds were sowed on MS medium supplemented without or with different concentrations of ABA. The germination percentage and the greening rate were scored. All the data represent the means of three independent experiments ± SD. * represent the significant differences at *p* < 0.05; ** represent *p* < 0.01; *** represent *p* < 0.001 (Student’s *t*-test).

To study the response of *bag2* and *bag6* single and *bag2 bag6* double mutants to the plant stress hormone ABA, these mutants and WT seeds were sown on Murashige and Skoog (MS) medium supplemented with different concentrations of ABA to compare the germination and greening rate between WT and mutants lines. Without ABA, the mutant seeds showed a slightly higher germination rate than that of the WT, and when ABA was applied, much higher germination and greening rates were observed in the *bag2* and *bag6* single and *bag2 bag6* double mutants (Figure 4). When comparing germination and greening rates on 0.75 μM ABA, we found a significant difference: WT germination rate (72%) and greening rate (46%), while *bag2* 75% and 84%, *bag6* 79% and 88% and *bag2 bag6* 81% and 91%, respectively (Figure 4B,C). Similarly, when compared on 1 μM ABA, WT exhibited 57% of seeds germinated and after 7 days a 29% greening rate was found, but the *bag2* single mutant exhibited 65% germination while a 49% greening rate was observed; *bag6* also showed higher germination and greening rates than the WT (i.e., 68% and 55%, respectively) (Figure 4B,C). Interestingly, the *bag2 bag6* double mutant’s germination rate was higher than each single mutant and WT, which are 75% and 71%, respectively (Figure 4B,C). Finally, when compared on 2 μM ABA, WT exhibited 37% germination and a 24% greening rate, while *bag2* exhibited 50% and 36%, *bag6* 55% and 36% and *bag2 bag6* 62% and 49%, respectively (Figure 4B,C). Thus, these mutants appeared to enhance the ABA tolerance of Arabidopsis during germination and the early vegetative growth period.

**Figure 5:**
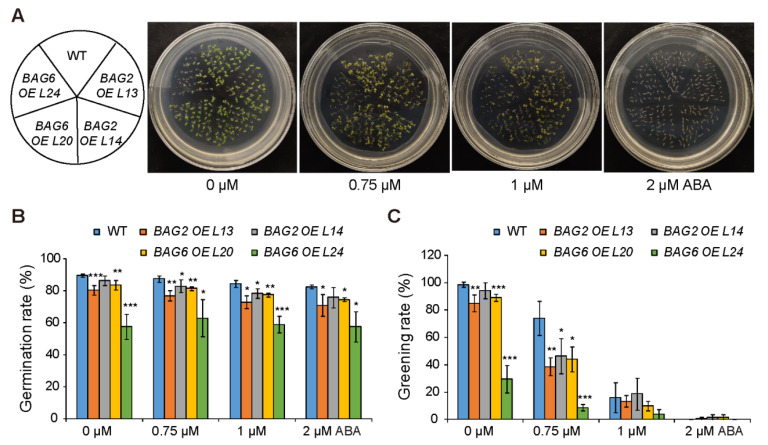
*AtBAG2* and *AtBAG6* overexpression lines are more sensitive to ABA than the WT. (**A**–**C**) Seed germination rate and greening rate of WT, *AtBAG2* and *AtBAG6* overexpression lines. Sterilized seeds were sowed on MS medium supplemented without or with different concentrations of ABA. The germination percentage and the greening rate were scored. Shown are means ± SD. * represent the significant differences at *p* < 0.05; ** represent *p* < 0.01; *** represent *p* < 0.001 (Student’s *t*-test).

To further analyze the role of *AtBAG2* and *AtBAG6* genes in Arabidopsis responses to ABA, we generated transgenic lines over expressing the *AtBAG2* and the *AtBAG6* gene (Figure S5A–D). Without ABA, seeds of the *AtBAG2* and *AtBAG6* overexpression (OE) lines showed a slightly lower germination rate and greening rate than the WT (Figure 5). When ABA was applied, much lower greening rates were observed in the *AtBAG2* and *AtBAG6* OE lines (Figure 5). When comparing germination and greening rates on 0.75 μM ABA, both OE lines and WT reduced significantly: WT germination rate (87%) and greening rate (74%), while *AtBAG2* OE L13 (77%) and (38%), *AtBAG2* OE L14 (83%) and (46%), *AtBAG6* OE L20 81% and 44%, *AtBAG6* OE L24 63% and 8% respectively (Figure 5B,C). Similarly, when compared on 1 μM ABA, WT germination rate and greening rate 84% and 16%, but *AtBAG2* OE L13 73% and 13%, *AtBAG2* OE L14 78% and 18%, *AtBAG6* OE L20 77% and 10%, and *AtBAG6* OE L24 59% and 4%, respectively (Figure 5B,C). Finally, when 2 μM ABA is applied, WT exhibited 82% and 0% germination and greening rates, *AtBAG2* OE L13 71% and 1%, *AtBAG2* OE L14 76% and 1%, *AtBAG6* OE L20 74% and 1%, and *AtBAG6* OE L24 57% and 0%, respectively (Figure 5B,C). 

Collectively, these data demonstrate that mutation of *AtBAG2* and *AtBAG6* genes enhances the ABA tolerance of Arabidopsis during germination and the early vegetative growth period, whereas their overexpression reduced germination speed and growth capability.

**Figure 6:**
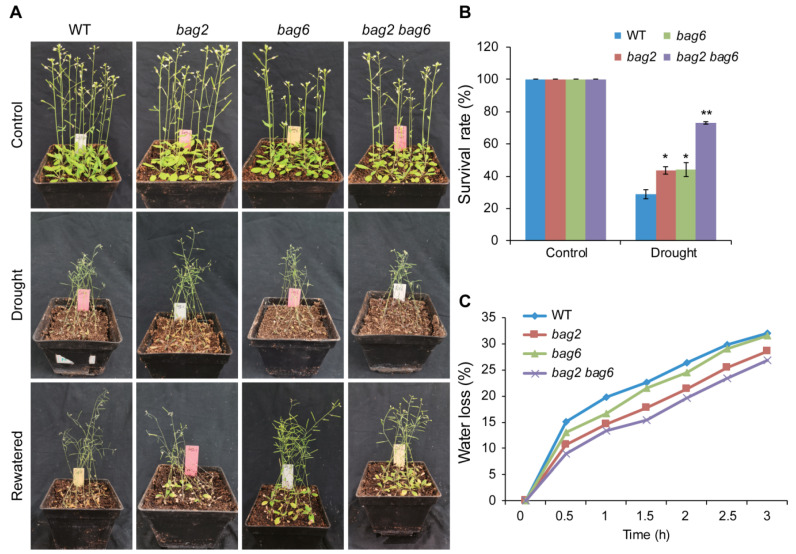
*bag2* and *bag6* mutants are more tolerant to drought. (**A**) Phenotypes of WT, *bag2* and *bag6* single, and *bag2 bag6* double mutant plants treated with drought stress. Three-week-old plants were subjected to drought stress for 14 days and re-watered for 3 days; (**B**) the survival rate of WT, *bag2*, *bag6*, and *bag2 bag6* plants after 3 days of re-watering following the 14 days drought treatment. Values are means ± SD (*n* = 5 independent experiments, 108 plants for each genotype in each independent experiment were used for analysis); (**C**) Water loss in WT, *bag2*, *bag6*, and *bag2 bag6* plants. Leaves from 3-week-old plants were collected and subjected to water loss at different time intervals. All the data represent the means ± SD of five independent experiments. * represent the significant differences at *p* < 0.05; ** represent *p* < 0.01 (Student’s *t*-test).

To study drought stress tolerance, 3-week-old WT and mutant plants were used, and 9 plants per pot were sown and 12 pots were prepared for each genotype (108 plants in total for each genotype). After withholding water for 14 days, almost all the WT and mutant plants were wilted and near to death (Figure 6A). Then, the plants were re-watered for three days. On the third day a significant difference was noticed between WT and mutant plants (Figure 6A); *bag2* showed 43%, *bag6* showed 44%, and *bag2 bag6* showed the highest survival rate at 73%, while the WT showed a lower survival rate at 29 % (Figure 6B). To further verify these results, we performed water loss experiments with different time intervals. Consistently, the water loss in mutant plants’ rosette leaves was slower than that of the WT (Figure 6C). These results suggest that *BAG2* and *BAG6* play a negative role in Arabidopsis responses to ABA and drought treatments.

### 2.4. Mutation of BAG2 and BAG6 in Arabidopsis Compromises Tolerance to Heat Stress

**Figure 7:**
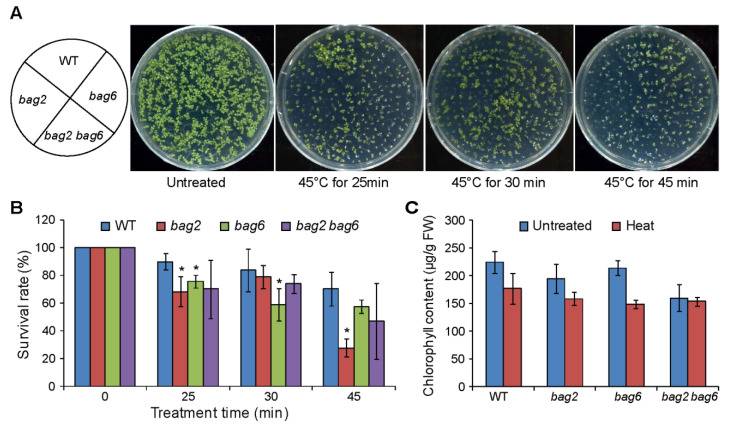
*bag2* and *bag6* mutants are hypersensitive to heat stress. (**A**) Phenotype of seedlings treated with heat stress; (**B**) Survival rates of WT, *bag2*, *bag6*, and *bag2 bag6* mutants under heat stress. Five-day-old seedlings were placed at 45 °C for 25, 30, and 45 min and then resumed growth at 22 °C; (**C**) Chlorophyll content measured from seedlings grown on normal growth conditions and after 45 °C heat stress. The data represent the means ± SD of two independent experiments. * represent the significant differences at *p* < 0.05 (Student’s *t*-test).

Our *ProBAG2:GUS* and *ProBAG6:GUS* expression results and previously reported RT-PCR/RT-qPCR and proteomics results show that *BAG2* and *BAG6* genes are upregulated during heat stress [17,19,28]. Thus, we studied the effect of heat stress on *bag2* and *bag6* single and *bag2 bag6* double mutants. The results showed that the *bag2* and *bag6* single and *bag2 bag6* double mutants are hypersensitive to heat stress compared to WT seedlings (Figure 7). After being treated at 45 °C for 25 min, the survival rates of WT, *bag2*, *bag6*, and *bag2 bag6* were 90%, 68%, 54%, and 70%, respectively; after being treated at 45 °C for 30 min, their survival rates were 84%, 79%, 59%, and 74%, respectively; and after being treated at 45 °C for 45 min, their survival rates were 70%, 28%, 57%, and 47%, respectively (Figure 7B). We also measured chlorophyll content in the mutants and WT on control untreated seedlings (22 °C) and 45 °C heat treated seedlings. Total chlorophyll levels decreased as a consequence of the heat treatment both in WT and mutants, but the mutants retained less chlorophyll (Figure 7C). These results indicate that mutation of *BAG2* and *BAG6* compromises Arabidopsis tolerance to heat stress.

**Figure 8:**
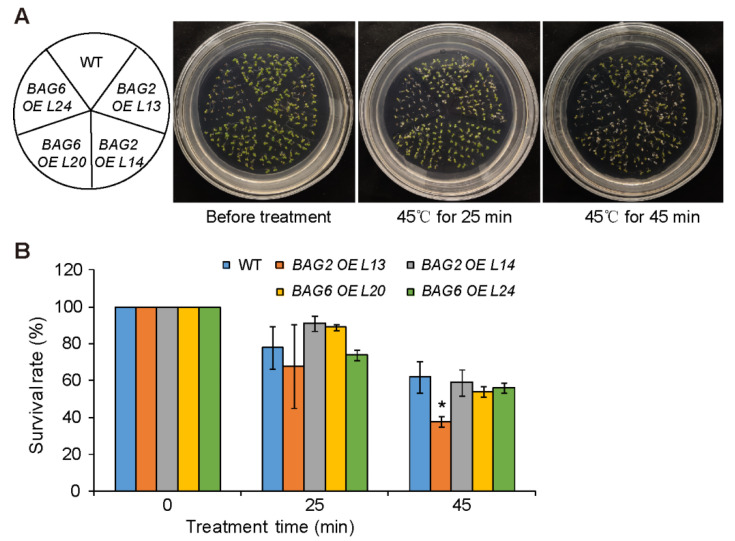
*AtBAG2* and *AtBAG6* OE lines display no significantly enhanced tolerance to heat stress. (**A**) Phenotypes of seedlings treated with heat stress; (**B**) Survival rates of WT, *AtBAG2* and *AtBAG6* OE lines under heat stress. Five-day-old seedlings were placed at 45 °C for 25 and 45 min and then resumed growth at 22 °C. Shown are means ± SD. * represent the significant differences at *p* < 0.05 by Student’s *t*-test.

We used the *AtBAG2* and *AtBAG6* OE lines to further analyze the role of *AtBAG2* and *AtBAG6* genes in Arabidopsis responses to heat stress. After being treated at 45 °C for 25 min, the survival rates of WT, *AtBAG2* OE L13 and L14, and *AtBAG6* OE L20 and L24 were 78%, 68%, 91%, 89%, and 74%, respectively; and after being treated at 45 °C for 45 min, their survival rates were 62%, 38%, 59%, 54%, and 56%, respectively (Figure 8). These results indicate that *AtBAG2* and *AtBAG6* overexpression does not significantly enhance Arabidopsis tolerance to heat stress. 

### 2.5. Stress- and ABA-Related Genes Are Differentially Regulated in the bag2, bag6, and bag2 bag6 Mutants Compared to WT

**Figure 9:**
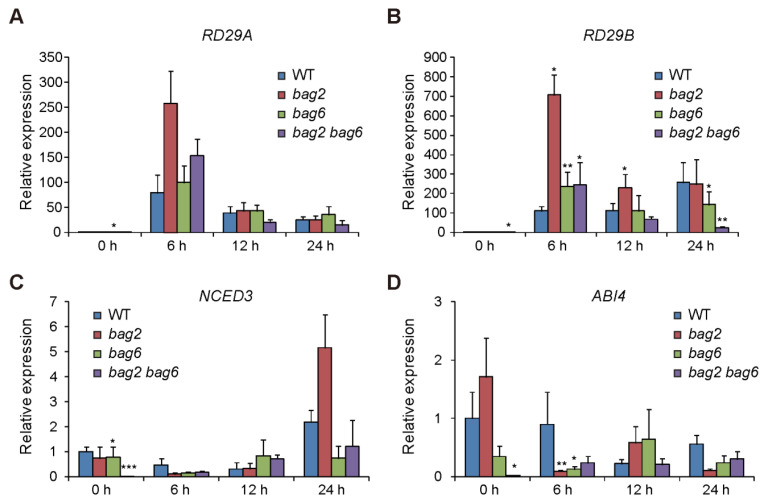
The relative expression of stress- and ABA-related genes. Transcription levels of *RD29A* (**A**)*, RD29B* (**B**), *NCED3* (**C**), and *ABI4* (**D**) genes in WT and *bag2*, *bag6*, and *bag2 bag6* mutants were examined under ABA treatment for 0, 6, 12, and 24 h. Values are means ± SD of three replicates. * represent the significant differences at *p* < 0.05; ** represent *p* < 0.01; *** represent *p* < 0.001 (Student’s *t*-test).

To further investigate the role of *BAG2* and *BAG6* in Arabidopsis responses to ABA, heat and drought treatment, we performed RT-qPCR analysis on several stress- and ABA-related genes such as *RD29A* and *RD29B* (stress related genes), *NCED3* (ABA biosynthesis gene), and *ABI4* (ABA response gene) [33,34,35,36,37,38]. We found that after ABA treatment the expression of *RD29A* and *RD29B* genes was upregulated in WT and *bag2*, *bag6*, and *bag2 bag6* seedlings, but the mutants showed more induction compared to the WT, particularly at 6 h of ABA treatment (Figure 9A,B). Whereas expression of *NCED3* and *ABI4* genes was downregulated by ABA treatment in WT and *bag2*, *bag6*, and *bag2 bag6* seedlings with the mutants showed more inhibition compared to the WT, particularly at 6 h of ABA treatment (Figure 9C,D). Interestingly, without ABA treatment the *bag6* and *bag2 bag6* seedlings already displayed lower transcription levels of the *NCED3* and *ABI4* genes compared to the WT (Figure 9C,D). This finding is consistent with the seed germination phenotype of these mutants. These results further support the ABA and drought phenotypic data.

### 2.6. ROS Accumulation in WT and Mutant Plants

**Figure 10:**
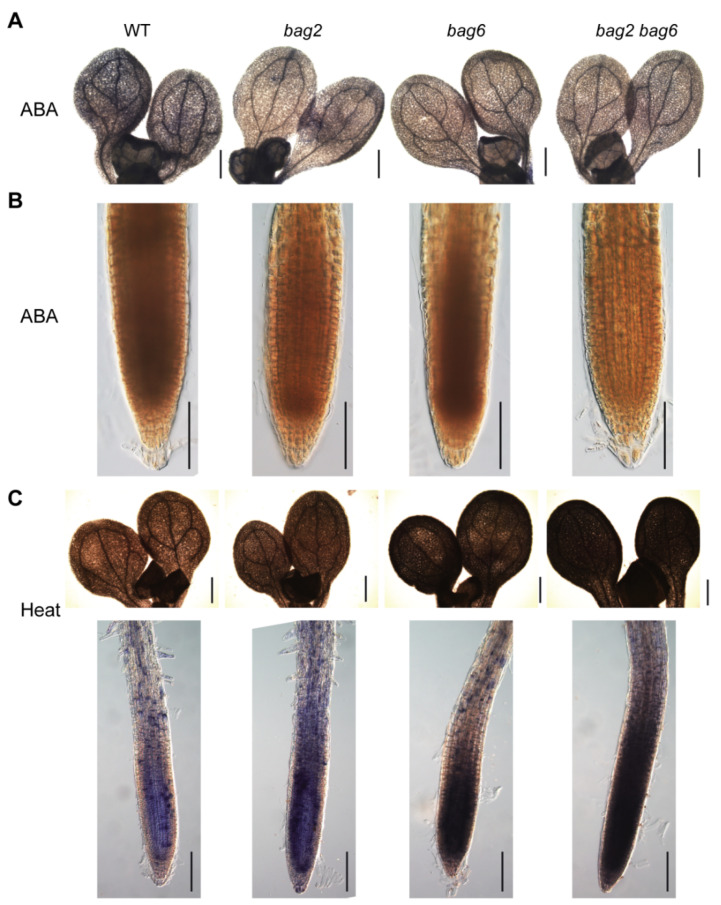
*BAG2* and *BAG6* mutation affects the ROS levels after ABA and heat treatment in Arabidopsis seedlings. Five-day-old seedlings were transferred to 1/2 MS agar plates supplemented with 20 μM ABA for 6 h or were treated with heat stress (37 °C) for 3 h, then were stained with NBT (staining for O_2_^–^) (**A**,**C**) or DAB (staining for H_2_O_2_) (**B**). At least three independent experiments were performed for each analysis and 15 seedlings of each genotype were examined. Bars = 400 μm in (**A**) and in the shoot images in (**C**) and 100 μm in (**B**) and in the root images in (**C**).

The accumulation of H_2_O_2_ and superoxide anion (O_2_^–^) was detected using 3,3-diaminobenzidine (DAB) and nitro blue tetrazolium (NBT) staining in seedling shoots and roots. Upon 20 μM ABA treatment, the cotyledons and root of the mutant seedlings displayed lighter color than that of the WT, which indicates lower accumulation of O_2_^–^ and H_2_O_2_, respectively, in the mutant seedlings (Figure 10A,B). However, upon heat stress treatment, the mutants exhibited higher accumulation of O_2_^–^ than the WT (Figure 10C). Finally, DAB staining was also applied to drought stressed rosette leaves of WT and mutant plants. We obtained the same results, such that leaves of mutant lines accumulated lower H_2_O_2_ than that of the WT (Figure S6). Combined, these results indicate that mutant lines had a lower accumulation of ROS when treated with ABA and drought while having higher ROS accumulation when treated with heat stress. These results correspond well with the respective drought- and heat-tolerance phenotypes of these mutants.

## 3. Discussion

In plants, especially in Arabidopsis, BAG proteins are a topic of interest and they mediate multiple abiotic, biotic and developmental processes. Actually, most research on plant BAGs so far has focused on their functions in abiotic and biotic stress tolerance. The expression patterns of *AtBAG* genes and their roles during plant development remain largely unknown. In this study, we analyzed the expression patterns of *AtBAG2* and *AtBAG6* genes during development by using *ProBAG2:GUS* and *ProBAG6:GUS* transgenic lines. Interestingly, the results show that both *AtBAG2* and *AtBAG6* genes are expressed in various tissues with overlapping or specific expression patterns during the whole developmental process. For example, *ProBAG6:GUS* is expressed in all of the root tissues with stronger expression in columella root cap cells and root vascular tissue, but *ProBAG2:GUS* is only expressed in the root vascular tissue. The root cap surrounds and protects the root tip meristem and receives and transmits environmental signals to the growing root [39]. As the root grows, the outermost layers of the root cap cells undergo cell death [39]. The strong expression of *AtBAG6* in the columella root cap cells implies that *AtBAG6* might play a role in the PCD of root cap cells. In shoots, both *ProBAG2:GUS* and *ProBAG6:GUS* are strongly expressed in rosette leaves and cauline leaves with the strongest signal in the vasculature strands. The developmental process of vasculature formation involves cell differentiation and PCD [40]. The strong expression of *AtBAG2* and *AtBAG6* genes in the vascular tissues imply that they may have a role in the formation of vasculature strands. Detailed examination of vascular tissue development in the *bag2* and *bag6* mutants and *AtBAG2* and *AtBAG6* OE plants under normal and stressed growth conditions will provide some clue.

Mutant analysis showed that *bag2* and *bag6* single mutants and *bag2 bag6* double mutant plants are slightly larger than the WT, and they show no developmental defect. These results suggest that the *AtBAG2* and *AtBAG6* genes and other *AtBAG* genes may have redundant and specific functions during Arabidopsis development. Analyzing the other *AtBAG* genes expression patterns and generating multiple *atbag* mutants, by crossing or by using CRISPR/Cas9 gene editing methods, will provide more information for understanding the role of the *AtBAG* family genes during Arabidopsis development.

Previous RT-qPCR and Northern blot analysis had shown that *AtBAG2* transcript levels were significantly upregulated by ABA and salt treatment and *AtBAG6* transcript levels were significantly upregulated by ABA, SA, H_2_O_2_ and heat treatment [10,17,19,28]. Our *ProBAG2:GUS* and *ProBAG6:GUS* expression results show that they both are upregulated by heat, salt and osmotic treatment and by stress-related plant hormones including ABA, SA and ACC treatment, and *ProBAG6:GUS* is additionally induced by JA. These expression results suggest that the *AtBAG2* and *AtBAG6* genes may be involved in Arabidopsis responses to multiple environmental stresses. Indeed, our stress and hormone treatments results show that the *bag2* and *bag6* single mutants and *bag2 bag6* double mutant seeds germination are less sensitive to ABA treatment and their plants are more tolerant to drought stress than the WT, but they are more sensitive to heat stress. By contrast, *AtBAG2* and *AtBAG6* OE seed germination is more sensitive to ABA treatment. Interestingly, in ABA and drought treatments the *bag2 bag6* double mutant shows more tolerance than either of the single mutants, suggesting an additive effect of these two genes in Arabidopsis responses to ABA or drought stress treatments. There is some discrepancy on *atbag6* seedlings response to heat stress among the previously published results. Nawkar et al. (2016) showed that *atbag6* (SALK_004760) seedlings were hypersensitive to heat stress treatment (45 °C for 25, 30, and 45 min) compared to the WT [17]. However, Echevarría-Zomeño et al. (2016) found that *atbag6* (SALK_047959 and SALK_015968) seedlings displayed mild tolerance to heat stress (45 °C for 30 min) compared to the WT seedlings [28]. In heat-induced acclimation experiments (38 °C for 2 h, followed by 45 °C for 1–3 h), Fu et al. (2019) showed that *atbag6* (SALK_047959C and SALK_073331C) seedlings exhibited the same thermotolerance phenotypes as the WT, but that they could enhance the thermotolerance of *fes1a* (Fes1A is a co-chaperone of the HSP70 protein) mutant [19]. Although Nawkar et al. (2016) and Echevarría-Zomeño et al. (2016) used same heat stress treatment conditions, they used a different *atbag6* T-DNA insertional mutant [17,28]. We used the same *atbag6* (SALK_004760) mutant and heat treatment conditions as Nawkar et al. (2016) and obtained similar results. Therefore, the discrepancy on *atbag6* thermotolerance might be due to a different T-DNA insertional mutant used for the heat stress treatment. We also performed heat stress treatment on *AtBAG2* and *AtBAG6* OE lines (selected two OE lines for each gene). After being treated at 45 °C for 25 min, one *AtBAG2* OE line and one *AtBAG6* OE line exhibited a slightly higher survival rate compared to the WT, but the other line did not. Whereas after being treated at 45 °C for 45 min, both *AtBAG6* OE lines and one *AtBAG2* OE line showed same thermotolerance phenotypes as the WT but one *AtBAG2* OE line displayed a significantly lower survival rate. In the future, more OE lines for each gene should be selected to perform heat stress treatments in order to obtain convincing conclusions.

Although our RT-qPCR analysis results showed that several stress- and ABA-related genes are differentially expressed in *bag2*, *bag6*, and *bag2 bag6* seedlings compared to the WT, the underlying molecular mechanism remains to be unveiled. The molecular mechanism of how AtBAG6 mediates fungal resistance has been revealed recently [27]. Fungal infection triggers AtBAG6 cleavage by an aspartyl protease and the processed AtBAG6 activates autophagy and plant defense [27]. Whether a similar molecular mechanism could be responsible for AtBAG6-mediated abiotic stress tolerance awaits future study. *atbag7* seedlings also are sensitive to heat treatment [20,31]. The underlying molecular mechanism responsible for AtBAG7 heat stress protection was revealed by in silico analysis of its amino acid sequence, by examination of its localization under normal and stress conditions, and by screening AtBAG7-interacting proteins [31]. In the future, similar experiments can be performed on AtBAG2 and AtBAG6 to provide some insight into understanding their functional mechanisms during plant responses to abiotic stress. 

## 4. Materials and Methods 

### 4.1. Plant Materials and Growth Conditions

The T-DNA insertion mutants *bag2* (SALK_030295) and *bag6* (SALK_004760) are in Columbia-0 (Col-0) background. These mutants were obtained from the Arabidopsis Biological Resource Center (ABRC), and the double mutant was obtained by crossing *bag2* and *bag6*. T-DNA insertion was confirmed by RT-PCR analysis. Primers used in this experiment are listed in Supplementary Table S1. Seeds were surface sterilized in 70% ethanol for 5 min and 1% Clorox bleach for 10 min, stratified for 3 days at 4 °C. The seeds were plated on solidified Murashige and Skoog (MS) medium and vertically grown at 22 °C under 16 h light/8 h dark conditions. For ABA treatment experiments, all the single, double mutant, and WT seeds were surface sterilized and, after 3 days stratification, sown on plates supplemented with or without ABA. For drought treatments, the stratified seeds were grown vertically on MS plates for seven days, and then were transferred to soil (75% soil and 25% vermiculite) for more 14 days to grow. Nine plants per pot were grown. During each independent experiment, we grew 108 plants in total for each genotype.

### 4.2. Vector Construction and Generation of Transgenic Plants

To create the *ProBAG2:GUS* construct, the 1886 bp sequence upstream of the *BAG2* gene start codon was PCR-amplified. The resulting fragment was inserted upstream of the *GUS* reporter gene in the pGreenII0229-GUS vector via *Xho*I and *Kpn*I sites [41]. Similarly, *ProBAG6:GUS* was also created for this study, and the 1884 bp sequence upstream of the *BAG6* gene start codon was PCR-amplified. The resulting fragment was inserted upstream of the *GUS* reporter gene in the pGreenII0229-GUS vector via *Xho*I and *Kpn*I sites [41]. Primer sequences used in this experiment are available in Supplementary Table S1. These constructs were transformed into *Agrobacterium tumefaciens* strain C58C1 (pMP90/pJICSa-Rep). The Arabidopsis transgenic plants were generated using floral dip method [42].

### 4.3. RT-PCR Analysis

RT-PCR was performed to test the transcription levels of the *BAG2* and *BAG6* gene in mutant or WT plants. Total RNA was extracted from 7-day-old seedlings using Trizol reagent (TransGen Biotech, Beijing, China). The first-strand cDNA was synthesized using Easy Script First-Strand cDNA Synthesis Super Mix (TransGen Biotech, Beijing, China). The *ACTIN2* gene was used as an internal control. The primer sequences used in this experiment are listed in Supplementary Table S1. 

### 4.4. GUS Histochemical Staining

For GUS staining, various tissues of *ProBAG2:GUS* and *ProBAG6:GUS* transgenic plants were incubated for 12 h at 37 °C in GUS staining buffer as previously described [43]. After staining, the tissues were cleared in chloral hydrate: distilled water: glycerol (8:3:1, *w*:*v*:*v*). Samples were visualized with microscopy (Olympus BX63, Tokyo, Japan). 

### 4.5. Drought and ABA Treatments

The *bag2* and *bag6* single and *bag2 bag6* double mutants and WT seeds were harvested at the same time and used for ABA treatments. In brief, surface-sterilized seeds were sown on MS medium plates supplemented without or with different concentrations of ABA (Sigma-Aldrich, St. Louis, MO, USA) as indicated. Plates were stratified at 4 °C in the dark for 72 h and transferred to a growth incubator for normal conditions (16 h light/8 h dark, 22 °C). For quantification of the percentage of seedlings with green cotyledons, seeds were sown on MS medium supplemented different concentrations of ABA and analyzed on the indicated days after stratification as previously described [44]. Pictures were scanned using HP LaserJet P1108 scanner.

For the drought tolerance test, 3-week-old plants grown in soil with sufficient water were treated with natural drought (water was withheld). After 14 days without watering, the drought-treated plants were re-watered and recovery was checked after 3 days. Drought experiments were repeated three times, and at least 9 plants for each individual genotype were used per experiment. 

### 4.6. Heat Treatment and Measurement of Chlorophyll Content

For heat treatment, 5-day-old WT, *bag2*, *bag6* and *bag2 bag6* seedlings were exposed to 45 °C for 45 min. After three days of recovery at 22 °C, the seedlings’ survival rate was counted [15]. For the measurement of chlorophyll content, mesophyll cells were spectrophotometrically measured as previously reported [45]. 

### 4.7. DAB and NBT Staining

DAB and NBT staining were performed as previously described [46]. The experiment was performed three times and each time 20 seedlings of each genotype were stained. Five-day-old seedlings were used in this experiment. 

### 4.8. Hormone Treatment

Hormonal treatment of the *ProBAG2:GUS* and *ProBAG6:GUS* seedling was performed as previously described [47]. 

### 4.9. RT-qPCR

For RT-qPCR analysis, of different stress related genes, we followed previously described protocol [48].

## 5. Conclusions

Results in the present study show that the *AtBAG2* and *AtBAG6* genes are broadly expressed in various tissues during Arabidopsis development. In addition, *AtBAG2* and *AtBAG6* expression is induced by abiotic stresses such as heat, salt and osmotic treatment and by stress-related plant hormones including ABA, SA, JA, and ACC. Seed germinations of *bag2* and *bag6* single and *bag2 bag6* double mutants are more tolerant to ABA treatment and their plants are more tolerant to drought stress but sensitive to heat stress. These findings suggest that *AtBAG2* and *AtBAG6* play important roles in *Arabidopsis* response to multiple abiotic stresses and hormones.

## Data Availability

Arabidopis mutant lines (SALK_030295, SALK_004760) were kindly provided by Arabidopsis Biological Resource Center (ABRC) and the double mutant was obtained by crossing *bag2* and *bag6*.

## Supplementary Materials

Supplementary materials can be found at https://www.mdpi.com/article/10.3390/ijms22115856/s1.

## Abbreviations

BAG	Bcl-2 associated athanogene
ABA	Abscisic acid
ACC	1-aminocyclopropane-1-carboxylic acid
JA	Jasmonate
SA	Salicylic acid
GA	Gibberellic acid
WT	Wild type
GUS	β-glucuronidase
PCD	programmed cell death
ROS	reactive oxygen species
