# Supplementary material for: The BAG2 and BAG6 Genes Are Involved in Multiple Abiotic Stress Tolerances in Arabidopsis Thaliana
Source: Int J Mol Sci. 2021 May 29;22(11):5856. doi: 10.3390/ijms22115856 (PMC8198428; doi:10.3390/ijms22115856)
Supplement: Supplementary file 1 [file ijms-22-05856-s001.zip › ijms-1180225-supplementary.pdf]

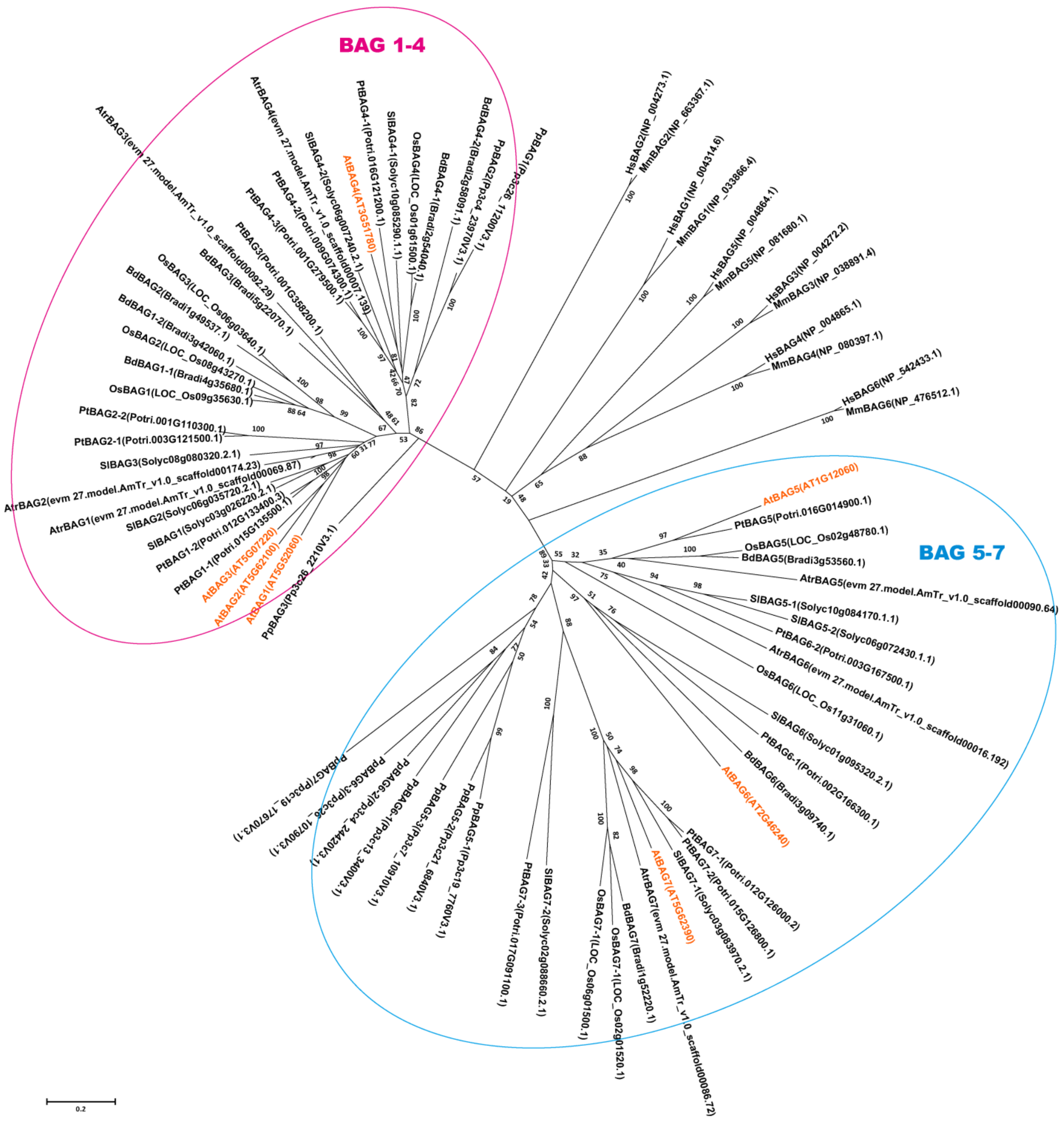

Figure S1. Phylogenetic analysis of BAG proteins in *Physcomitrella patens* (*Pp*), *Amborella trichopoda* (*Atr*), *Oryza sativa* (*Os*), *Brachypodium distachyon* (*Bd*), *Solanum lycopersicum* (*Sl*), *Populus trichocarpa* (*Pt*), *Arabidopsis thaliana* (*At*), *Homo sapiens* (*Hs*), and *Mus musculus* (*Mm*) by using the MEGA7 software.

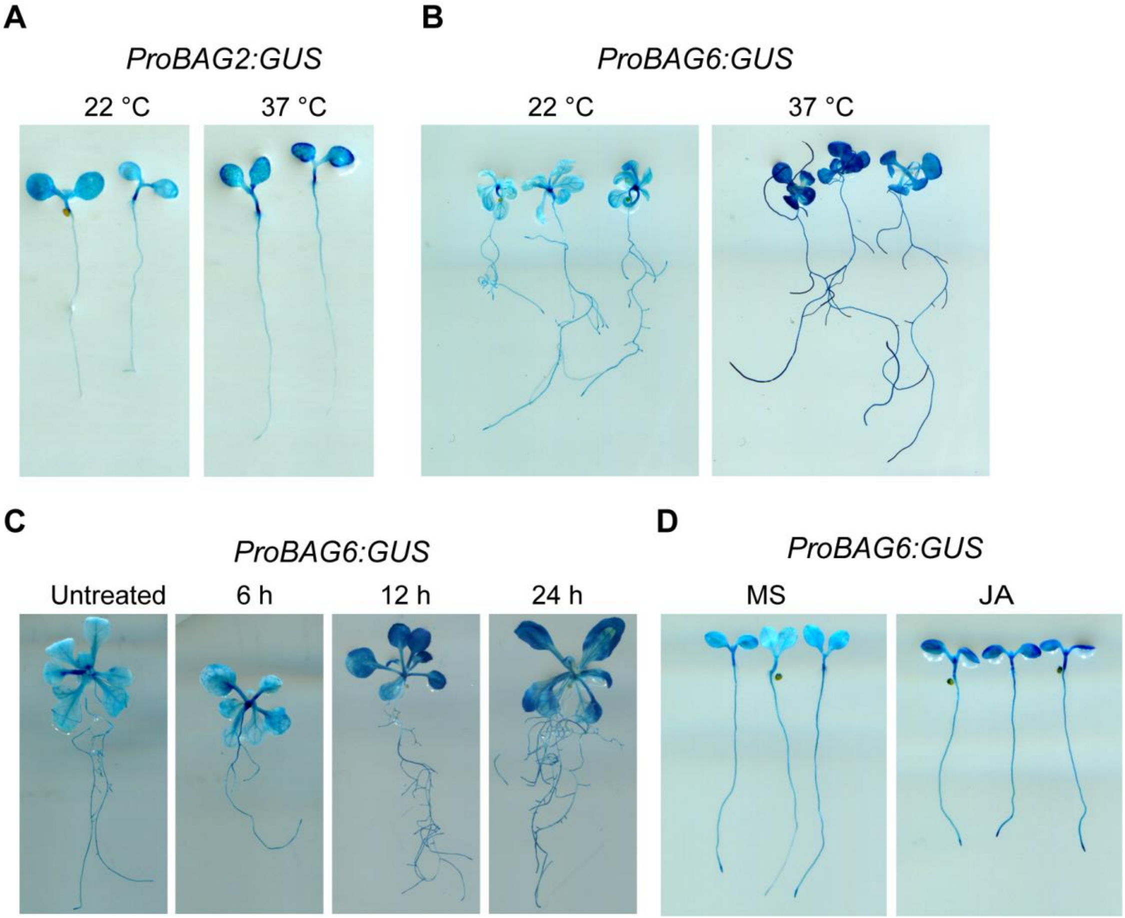

Figure S2. Histochemical GUS staining of *ProBAG2:GUS* and *ProBAG6:GUS* seedlings under a normal growth condition (22 °C) and after heat stress (37 °C), mannitol and jasmonic acid (JA) treatment. **A** and **B** GUS staining of 7-day-old *ProBAG2:GUS* and 12-day-old *ProBAG6:GUS* seedlings grown under a normal growth condition (22 °C) or after heat stress (37 °C) for 2 h. **C** GUS staining of 2-week-old *ProBAG6:GUS* transgenic plants treated without or with 300 mM mannitol for 6 h, 12 h and 24 h. **D** GUS staining of 7-day-old *ProBAG6:GUS* seedlings treated without or with 10 μM JA. Shown are representative images of three independent experiments.

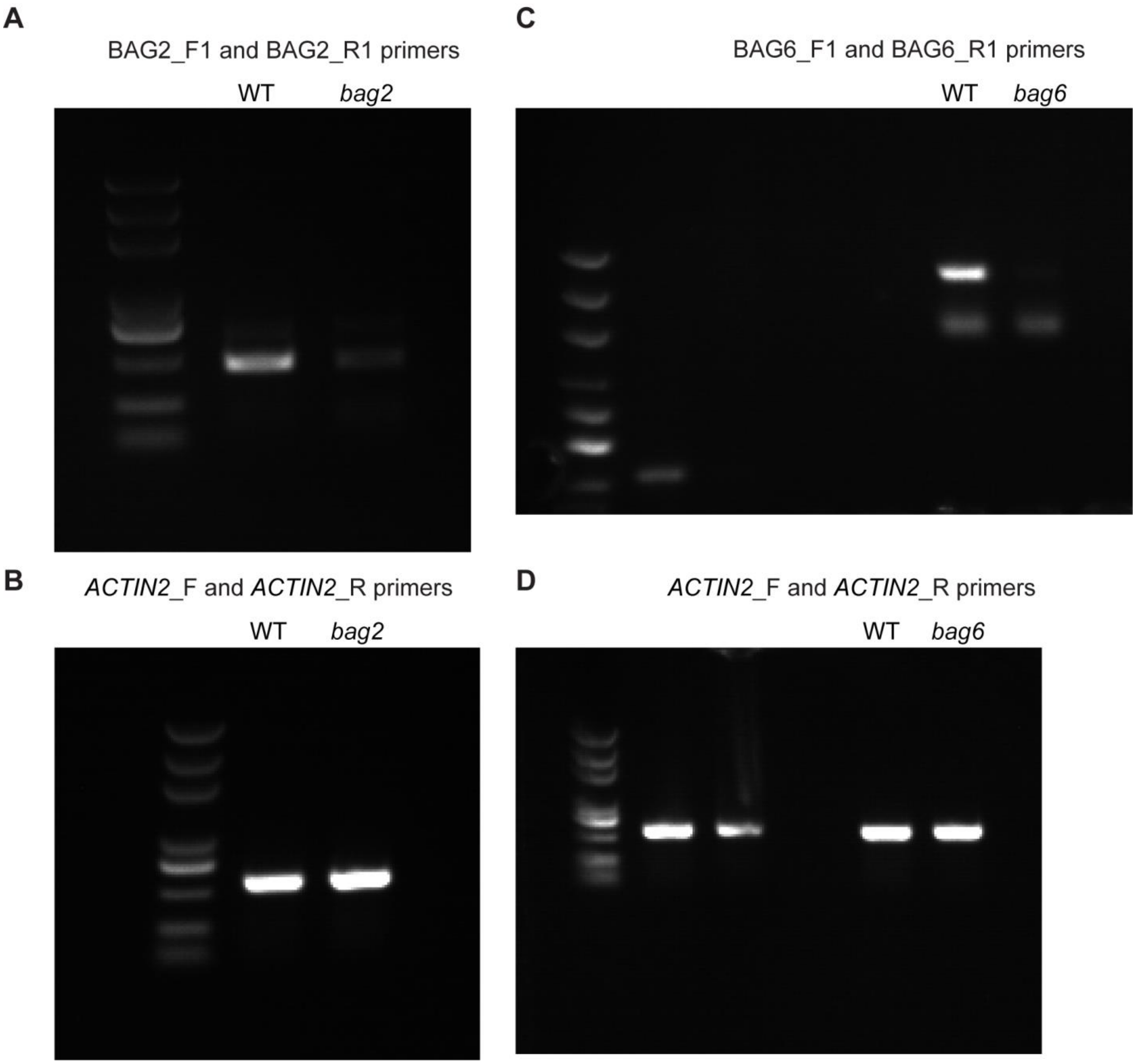

Figues S3. Transcription analysis of the *bag2* and *bag6* mutants.
a and **b** RT-PCR analysis of transcription levels of the *AtBAG2* (a) and the *ACTIN2* (b) genes in WT and the *bag2* mutants. **c** and **d** RT-PCR analysis of transcription levels of the *AtBAG6* (c) and the *ACTIN2* (d) genes in WT and the *bag6* mutants.

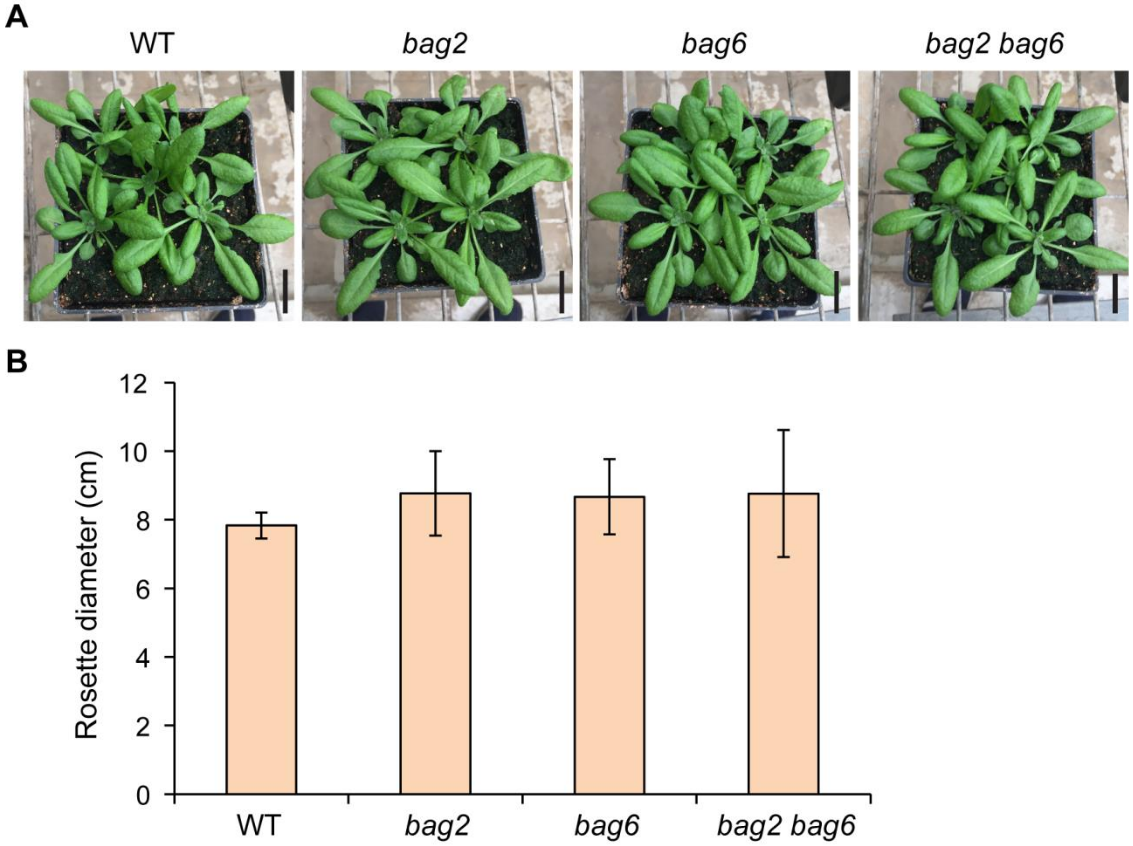

Figure S4. Phenotypes (A) and rosette diameter (B) of 4-week-old wild-type (WT), *bag2*, *bag6*, and *bag2 bag6* plants grown under a normal condition.

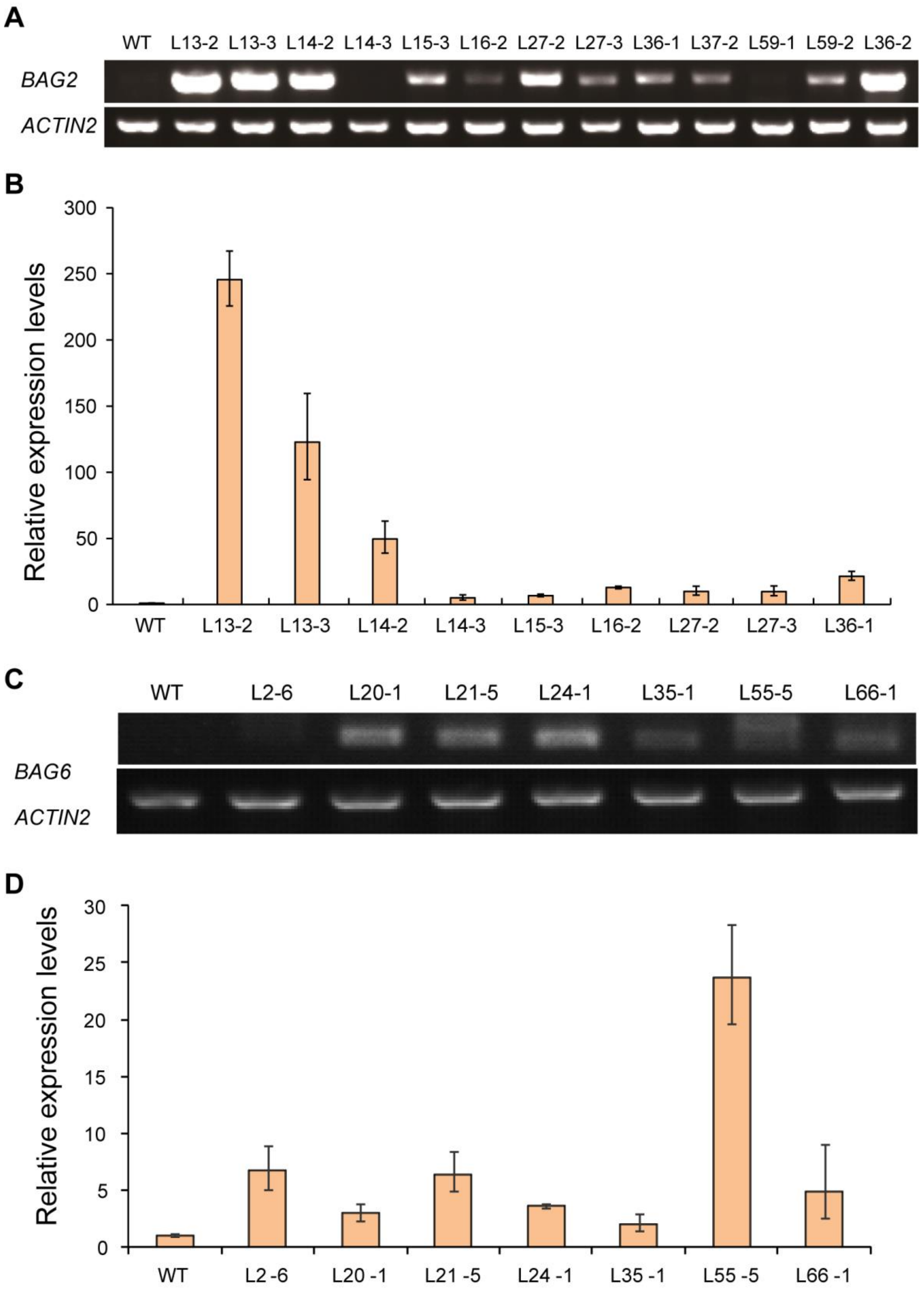

Figure S5. Transcription analysis of *AtBAG2* and *AtBAG6* overexpression lines. Three-week-old Arabidopsis rosette leaves were collected for RT-PCR (A, C) and RT-qPCR (B, D) to check the transcription levels of the *AtBAG2* and *AtBAG6* genes in WT and *AtBAG2* and *AtBAG6* overexpression transgenic lines.

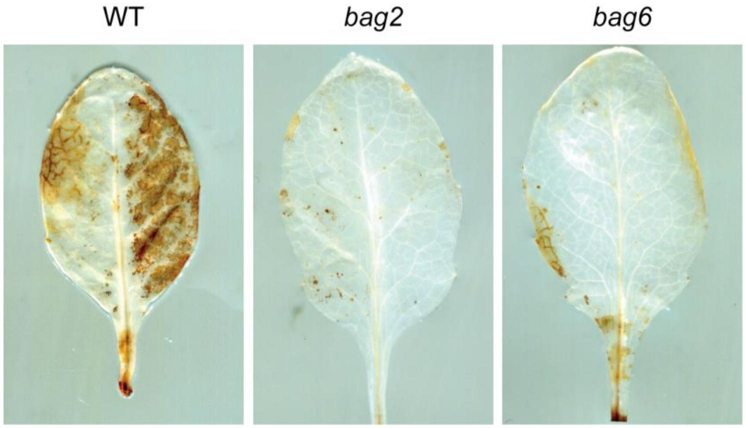

Figure S6. DAB staining of H_2O_2 accumulation in leaves from WT, *bag2* and *bag6* plants after two weeks drought treatment.

Table S1. List of Primers used in this study.

Name	Sequence (5′–3′)	Application
LBa1	TGGTTCACGTAGTGGGCCATC	T-DNA specific primer
BAG2_F1	GGAGGGAAAGTTGAAGAGAAGA	Genotyping *bag2* mutant
BAG2_R1	TGCTGGATGTTGACGACATGTT	
BAG6_F1	CGAGAATGATCTGGAGAGCAG	Genotyping *bag6* mutant
BAG6_R1	GCTGGTTATAGCTTCCCTAACC	
BAG2_F1	GGAGGGAAAGTTGAAGAGAAGA	RT-PCR
BAG2_R2	CGACCAATCACAAATCATATACA	
BAG6_F3	GCCTGTGTACATGGATCCATC	
BAG6_R1	GCTGGTTATAGCTTCCCTAACC	
ACT2_F	TGGGATGAACCAGAAGGATG	RT-PCR reference
ACT2_R	AAGAATACCTCTCTTGGATTGTGC	
RD29A_F	GAAGATGATGATGATGACGAGC	RT-qPCR
RD29A_R	TCAGTGGGTTTGGTGTAATCG	
RD29B_F	AGCAAGACCCAGAAGTTCAC	
RD29B_R	AACAATCTCCTCCGATGC	
NCED3_F	ACAGCCTCGTCCCTAAGTCT	
NCED3_R	GCCCTCCCTCCTAAAGTGAC	
ABI4-F	ACTCCAAGTTCCGTTACCGTG	
ABI4-R	GGGGTTAAGTTGAGCTGAGCA	
BAG2_qF	TTGAAGAAGAAGATGCAGGAGGAA	
BAG2_qR	CCTTGACGCCTCCTCGTAAA	
BAG6-qF	CCTGGCAACGGATTCTAAGC	
BAG6-qR	GGAATTGTTGTCGAGGAAGC	

TIP41_qF	GTATGAAGATGAACTGGCTGACAAT	RT-qPCR reference
TIP41_qR	ATCAACTCTCAGCCAAAATCGCAAG	
BAG2_F4_p	CGACACAAATGTCCCACCTTTA	*ProBAG2:GUS* cloning
BAG2_R4_p	TTCTTTATTAAGAGAGATAGAGAG	
BAG6_F4_p	TGGATATAAACAAGAGCACTGAT	*ProBAG6:GUS* cloning
BAG6_R4_p	CTTTAATCAAGATCACTTAACCAA	
